# Long-Term Positive and Negative Psychological Late Effects for Parents of Childhood Cancer Survivors: A Systematic Review

**DOI:** 10.1371/journal.pone.0103340

**Published:** 2014-07-24

**Authors:** Lisa Ljungman, Martin Cernvall, Helena Grönqvist, Brjánn Ljótsson, Gustaf Ljungman, Louise von Essen

**Affiliations:** 1 Psychosocial Oncology and Supportive Care, Department of Public Health and Caring Sciences, Uppsala University, Uppsala, Sweden; 2 Department of Clinical Neuroscience, Division of Psychology, Karolinska Institutet, Stockholm, Sweden; 3 Department of Women's and Children's Health, Pediatric Oncology, Children's University Hospital, Uppsala, Sweden; Davidoff Center, Israel

## Abstract

Increasing survival rates in childhood cancer have yielded a growing population of parents of childhood cancer survivors (CCSs). This systematic review compiles the literature on positive and negative long-term psychological late effects for parents of CCSs, reported at least five years after the child's diagnosis and/or two years after the end of the child's treatment. Systematic searches were made in the databases CINAHL, EMBASE, PsycINFO, and PubMed. Fifteen studies, published between 1988 and 2010, from 12 projects were included. Thirteen studies used quantitative methodology, one quantitative and qualitative methodology, and one qualitative methodology. A total of 1045 parents participated in the reviewed studies. Mean scores were within normal ranges for general psychological distress, coping, and family functioning. However, a substantial subgroup reported a clinical level of general psychological distress, and 21–44% reported a severe level of posttraumatic stress symptoms. Worry, disease-related thoughts and feelings, marital strains, as well as posttraumatic growth was reported. Several factors were associated with the long-term late effects, such as parents' maladaptive coping during earlier stages of the childs disease trajectory and children's current poor adjustment. Quality assessments of reviewed studies and clinical implications of findings are discussed and recommendations for future research are presented.

## Introduction

Over the past 20 years advancements in cancer treatment have increased survival rates for childhood cancer and the average five-year survival rate is approaching 80% [1]. Today one in 650 adolescents and young adults is a childhood cancer survivor (CCS), and roughly twice as many are parents of CCSs [2].

With survival being expected for most children struck by cancer understanding the long-term late effects of childhood cancer is important. The medical and physical late effects of childhood cancer are well documented and include cardiopulmonary, endocrine, musculoskeletal, and neurocognitive deficits as well as second malignancies [3], [4]. Regarding psychological late effects results have been mixed, with some studies reporting levels of psychological health comparable to controls [5], while others report that a subgroup of CCSs experience persistent, or even increasing, psychological distress from 10 years up to decades after diagnosis [6]. The literature on psychological health among parents of CCSs has reported elevated levels of psychological distress such as posttraumatic stress symptoms (PTSS), depression, anxiety, sleep disturbances, somatic symptoms, fear of recurrence, extensive worry, and fatigue [7]–[10]. In addition, a greater appreciation for life and changed values, conceptualized as posttraumatic growth (PTG), are reported [11], [12]. Barakat et al. [13] demonstrated that 90% of mothers of children previously treated for cancer report at least one positive consequence due to the child's cancer disease whereas almost half report four or more positive consequences.

Although psychological distress among parents of children with cancer has been shown to decrease as a function of time since the child's cancer diagnosis [14], a subgroup of parents reports a high level of psychological distress even after end of the child's treatment [7], [9]. After end of treatment parents have to handle the risk of relapse and report increased levels of worry and fear of recurrence [15], [16]. For some parents, the cancer experience causes marital strains, strains in relationships with the previously ill child and/or its siblings [14], [17], as well as economical and occupational difficulties [18], [19]. Parents of children with cancer face several challenges, both during and after the time when the child is ill, that may contribute to development and/or maintenance of psychological distress even years after end of the child's cancer treatment.

An increased understanding of the nature and prevalence of the long-term psychological late effects experienced by parents of CCSs, as well as the factors associated with/predicting these effects, is important to guide future research and clinical practice for this population. Clinical levels of psychological distress such as PTSS, anxiety, and depression has serious consequences, not only for the individual, but also for the society. For the individual the distress is associated with low quality of life, functional disability, parenting challenges, and increased risk for somatic disorders such as coronary heart disease [20]–[22]. For the society, the distress is associated with costs due to health care utilization, productivity loss, and sick leave [23].

To our knowledge, two systematic reviews on psychological late effects of childhood cancer for parents have been published. Wakefield et al. [10] reviewed studies on psychological effects during the first two years after end of the child's treatment and found that parents were at increased risk of PTSS, anxiety, and feelings of loneliness and uncertainty, but that such distress seemed to abate with time since end of treatment. Bruce [7] reviewed studies on self-rated PTSS and clinician-assessed posttraumatic stress disorder (PTSD) among parents of CCSs. A 10–44% prevalence of severe PTSS and a 27–54% life-time prevalence of PTSD were shown. However, in the review by Bruce [7] time varied from recently after, up to several years after end of the child's treatment, and reports for parents of long-term survivors were not presented separately. Recent research on long-term psychological late effects among CCSs [6], and survivors of cancer during adulthood [24], shows that the survivors may experience increased levels of psychological distress 10 years up to decades after diagnosis. It is an unanswered question if these findings also apply to significant others, such as parents.

In order to establish knowledge on the long-term cancer-related psychological late effects among parents of CCSs, studies reporting on these effects need to be summarized. To the best of our knowledge this systematic review is the first attempting to reach this end.

### Objectives

The main objective was to describe the nature and prevalence of the long-term psychological late effects of childhood cancer for parents of CCSs. We chose a well-established, however conservative, definition of CCSs, defining a CCS as a person who has completed treatment for cancer, who is at least five years post diagnosis [5], [6] and/or at least two years after end of treatment. Psychological late effects were broadly defined including psychological function and distress, coping, and relational functioning. The second objective was to summarize the findings on factors associated with/predicting the long-term psychological late effects identified in the reviewed studies.

### Review Questions

1. Do parents of CCSs report long-term negative psychological late effects? If so, what are the nature, prevalence, and clinical severity of these?
2. Do parents of CCSs report long-term positive psychological late effects? If so, what are the nature and prevalence of these?
3. Are parents of CCSs at increased risk for long-term negative psychological late effects in comparison to parents of children not struck by cancer?
4. Are there any factors that are associated with or predict long-term psychological late effects reported by parents of CCSs?

## Method

The review was conducted according to the Preferred Reporting Items for Systematic Reviews and Meta-Analyses Statement (PRISMA), consisting of an evidence-based set of items for conduction and reporting systematic reviews and meta-analyses. Objectives and methods were specified in advance, documented in a protocol, and registered at PROSPERO (21/12–2012, CRD42012003521).

### Identification of Studies

Inclusion criteria were: observational study using quantitative and/or qualitative methodology; published in the English language in a peer-reviewed journal during the last 30 years (1982–2012); and reporting psychological effects of childhood cancer for parents of CCSs diagnosed with cancer at the age of 0–18 years, who had completed treatment and was diagnosed at least five years prior to study participation and/or had completed treatment at least two years prior to study participation. Thus, only studies reporting on parents of children off cancer treatment were included. Studies were excluded if the sample included parents of children with shorter time intervals since diagnosis or end of treatment unless data were presented in a way that isolation of outcomes for the population meeting the inclusion criteria was possible.

A search strategy was developed and the following databases were searched: CINAHL, EMBASE, PsycINFO, and PubMed. Search strategies were adapted for each database using a combination of free text and controlled terms. Terms were chosen based on search terms used in a previous review conducted on a similar topic [10] and an inspection of the controlled terms in each data base. Terms used were: ‘After treatment', Long-term', ‘Neoplasms’, ‘Off-treatment’, ‘Parents’, ‘Post-treatment’, ‘Successful treatment’, ‘Survivors’, ‘Time’, and ‘Treatment complete’. Example of full electronic search strategy used in PubMed: ("Parents"[Mesh]) AND ("Neoplasms"[Mesh]) AND (("Survivors"[Mesh]) OR ("Time"[Mesh]) OR ("Long-term"[All Fields]) OR ("Post-treatment"[All Fields]) OR ("After treatment"[All Fields]) OR ("Successful treatment"[All Fields]) OR ("Off-treatment"[All Fields]) OR ("Treatment complete"[All Fields])). Reference lists of included studies were screened for additional studies not found via searches in databases.

### Study Selection

**Figure 1:**
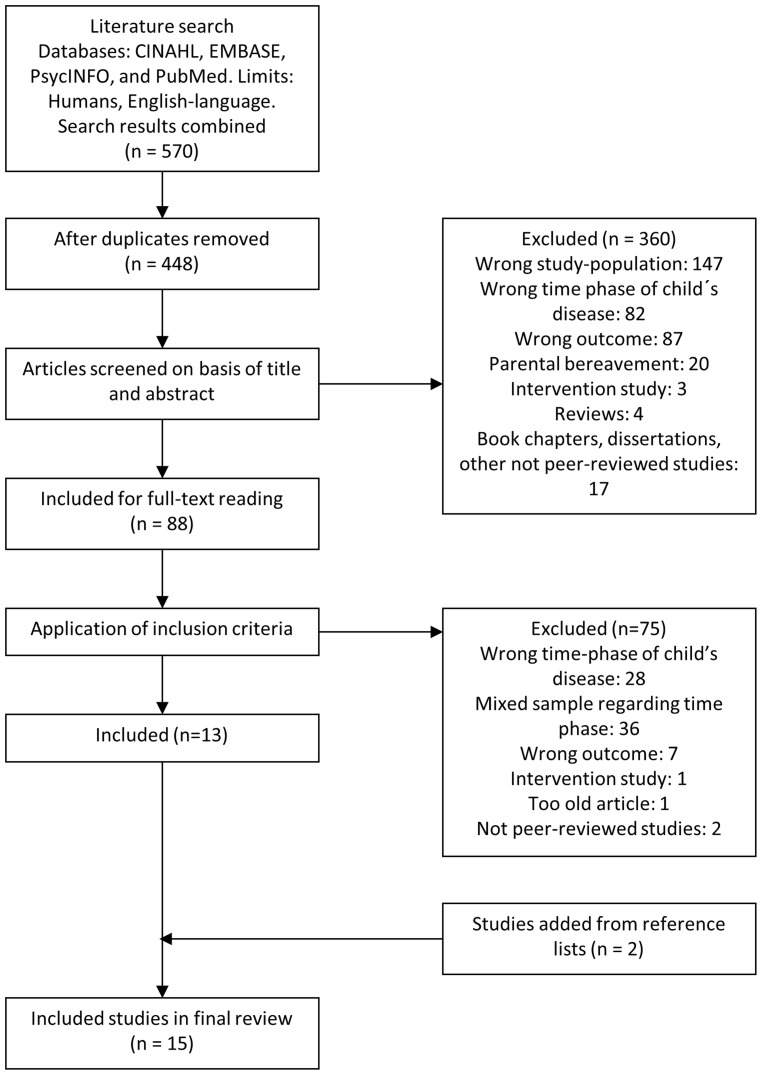
Flow Diagram of Included Studies.

570 studies were identified. After removing duplicates 448 remained. Titles and abstracts were independently screened by two authors (LL and MC). 360 studies were excluded as they did not meet the inclusion criteria. The 88 remaining studies were independently read in full text by two authors (LL and MC) to assess eligibility. When sufficient information to assess eligibility was not reported authors were contacted. Thirteen studies met the inclusion criteria. Two additional studies were identified through reference lists in included studies resulting in a total of 15 included studies. See flow diagram, Figure 1.

### Data Collection Process

We developed a data extraction sheet, pilot-tested it, and refined it accordingly. Pilot testing was made by extracting data from three studies excluded from the current review. Extracted data comprise: study origin; study design; parental sample (size, gender); child's characteristics (gender, age at study participation, diagnosis, age at diagnosis, time since diagnosis, time since completion of treatment); and outcomes comprising parental distress and adjustment, and factors associated with/predicting these.

### Assessment of Quality

A detailed analysis regarding the quality of the studies was conducted. The studies were evaluated using an aggregate of the quality criteria for observational studies developed by Leboeuf-Yde and Lauritsen [25], and the assessment tool QUALSYST which provides quality criteria for studies using quantitative and qualitative methodology [26]. Separate quality criteria were used for studies with quantitative and qualitative methodology respectively. Studies using quantitative methodology were assessed on 12 items comprising study design, method of subject selection, response rate, sample size, analytic methods, and whether conclusions were supported by the results (Table 1). Studies using qualitative methodology were assessed on 10 items comprising study design, connection to theoretical framework, sampling strategy, analytic method, use of verification procedure, reflexivity of the account, and whether conclusions were supported by the results (Table 2). Two authors independently assessed quality (LL and HG) with an inter-rater reliability of Cohen's Kappa = 0.79 (p<0.01). Consensus decisions were made to provide all studies with a final score on each quality criteria item.

**Table 1:** Quality Assessment of Studies using Quantitative Methodology.

	Quality assessment form item^a^														
First author, year	A	B	C	D	E	F	G	H	I	J	K	L	Study sum	Ratio^b^	Quality of study
Greenberg [29], (1989)	1	2	2	1	2	0	1	2	2	0	1	1	15	0,63	Mod
Hardy [38], (2008)	1	1	1	1	0	0	2	0	1	2	2	1	12	0,50	Mod
Kazak [37], (1994)	2	2	2	2	0	1	2	1	1	2	1	2	18	0,75	High
Kupst [32], (1995)	2	1	1	1	1	1	1	0	2	1	1	2	14	0,58	Mod
Kupst [31], (1988)	1	1	1	0	0	0	1	0	1	2	1	2	10	0,42	Low
Leventhal-Belfer [35], (1993)	1	1	0	1	0	1	1	0	0	0	1	1	7	0,29	Low
Maurice-Stam [39], (2008)	2	2	2	1	0	0	2	0	2	0	1	2	14	0,58	Mod
Michel [12], (2010)	2	1	2	1	1	1	2	0	2	2	1	2	17	0,71	Mod
Overholser [42], (1991)	1	1	1	1	1	0	1	0	1	0	1	2	10	0,42	Low
Ozono [34], (2010)	2	2	2	1	0	1	2	2	2	1	1	2	18	0,75	High
Ozono [33], (2007)	2	2	2	1	0	1	2	2	2	1	2	2	19	0,79	High
Quin [36], (2005)	1	2	1	1	1	1	2	2	0	0	1	1	13	0,54	Mod
Stuber [41], (1996)	2	2	2	1	0	1	2	2	1	1	2	2	18	0,75	High
Wijnberg-Williams [40], (2006)	2	2	2	1	0	1	2	1	2	2	1	2	18	0,75	High
Item sum	22	22	21	14	6	9	23	12	19	14	17	24			
Ratio^c^	0,79	0,79	0,75	0,50	0,21	0,32	0,82	0,43	0,68	0,50	0,61	0,86			
Quality of all studies on item	High	High	High	Mod	Low	Low	High	Low	Mod	Mod	Mod	High			

*Note.* Item description: A; question/objective sufficiently described, B; study design evident and appropriate (in addition to corresponding item in QUALSYST [26]: for 2 points (p), study primarily designed to assess prevalence), C; method of subject/comparison group selection described and appropriate, D; subject (and comparison group, if applicable) characteristics sufficiently described, E; response rate >90% = 2 p, 70–90% = 1 p, <70% = 0 p, F; reasons for nonresponse described AND non-responders described AND comparison of responders and non-responders  = 2 p, at least one of the criteria mentioned for F = 1 p, none of the criteria mentioned for F = 0 p, G; outcome measure(s) well defined and robust to measurement/misclassification bias, H; sample size appropriate (rule of thumb n>100 = 2 p, n>50–100 = 1 p and n<50 = 0 p), I; analytic methods described/justified and appropriate, J; estimate of variance reported for the main results, K; results reported in sufficient detail, L; conclusions supported by the results, Mod  =  moderate.

a Full information regarding scoring of item A, C, D, G, H, I, J, K, L, se corresponding item in QUALSYST [26]. Scoring of item B, E, F, see item description in note under table.

b Ratio between total score for each study and maximum total score. Ratio <0.5 was assessed as low quality, 0.5–0.75 as moderate quality, and >0.75 as high quality.

c Ratio between sum of item score and maximum item score. Ratio <0.5 was assessed as low quality, 0.5–0.75 as moderate quality, and >0.75 as high quality.

**Table 2:** Quality Assessment of Studies using Qualitative Methodology.

	Quality assessment form item^a^												
First author, year	A	B	C	D	E	F	G	H	I	J	Study sum	Ratio^b^	Quality of study
Greenberg [30], (1991)	1	1	1	1	1	1	0	0	1	0	7	0,35	Low
Quin [36], (2005)	1	2	2	1	2	1	1	0	1	0	11	0,55	Mod
Item sum	2	3	3	2	3	2	1	0	2	0			
Ratio^c^	0,5	0,75	0,75	0,5	0,75	0,5	0,25	0	0,5	0			
Quality of all studies on item	Mod	High	High	Mod	High	Mod	Low	Low	Mod	Low			

*Note.* Item description: A; question/objective clearly described, B; design evident and appropriate to answer study question, C; context for the study is clear, D; connection to a theoretical framework/wider body of knowledge, E; sampling strategy described, relevant and justified, F; data collection methods clearly described and systematic, G; data analysis clearly described, complete and systematic, H; use of verification procedure to establish credibility of the study, I; conclusions supported by result, J; reflexivity of the account, Mod  =  moderate.

a Full information regarding scoring, se corresponding item in QUALSYST [26].

b Ratio between total score for each study and maximum total score. Ratio <0.5 was assessed as low quality, 0.5–0.75 as moderate quality, and >0.75 as high quality.

c Ratio between sum of item score and maximum item score. Ratio <0.5 was assessed as low quality, 0.5–0.75 as moderate quality, and >0.75 as high quality.

Each study was provided a total score and a ratio between the study score and the possible maximum score. The total score for all studies on each item in the quality assessment was summarized and a ratio was calculated. Ratio <0.5 was assessed as low quality, 0.5–0.75 as moderate quality, and >0.75 as high quality.

### Data Synthesis

A synthesis of data was made with guidance from two sources. According to the Centre for Reviews and Dissemination [27] the aim of the synthesis in a systematic review is to draw results together, explore whether results are consistent across studies, and investigate possible reasons for any inconsistencies. Mays et al. [28] suggest a narrative synthesis to move beyond a summary of study findings to a synthesis where conclusions can be drawn within and across studies to generate new insights and reveal previously unknown patterns. Accordingly, a synthesis was made by a categorization of all extracted data and an analysis was made within each category and across all categories. Due to the low number of studies utilizing consistent measures or comparisons to a control group it was not possible to conduct a meta-analysis.

## Results

### Study Characteristics

See Table 3 for a presentation of the 15 studies included in the review. Data from three projects were reported twice [29]–[34]. In these cases data were extracted from both studies to include all reported outcomes, but participants were counted once and results considered as concerning one sample. Thirteen studies were based on quantitative methodology, one on quantitative and qualitative methodology, and one on qualitative methodology. Ten studies used a cross-sectional and five a cohort design. Two studies used a comparison group, one consisting of parents of children not struck by cancer. One study compared parents of survivors and parents of relapsed and diseased children within the cohort. Three studies used clinician-ratings and self-reports the remaining self-reports only. Sample sizes varied from 27 to 185 with a total of 1045 parents. In all studies but four both mothers and fathers were included; only mothers were included in one study and parental gender was not reported in three studies. In total, 624 mothers and 289 fathers participated in the reviewed studies. The children were diagnosed at 0 to18 years of age and their mean age at diagnosis ranged from 3.6 to 10.3 years (two studies [35], [36] did not provide specific information on the children's age at diagnosis). In 10 studies children with various diagnoses were included, in three studies only children with leukemia were included, and in two studies children with various diagnoses except brain tumors were included.

**Table 3:** Summary for Studies on Psychological Late Effects in Parents of Childhood Cancer Survivors.

First author, publication year and origin	Study design	Sample size	Dx	Age at Dx	Age of child at study start, mean (SD and range)	Time since Dx/Time since end of treatment, mean (SD and range)	Data collection	Results for late effects^a^	Results for associated/predicting factors^a^
Greenberg [29], (1989) USA	Quantitative, cross-sectional, and comparative. Comparison group: mothers of non-ill children.	N = 118 mothers of CCSs. Comparison group: N = 87.	Mixed	M = 3.6 yrs (range = 2 mths- 9 yrs) Comparison group: NR.	M = 12.5 yrs (range = 8–16 yrs) Comparison group: NR.	M = 8.8 yrs (range = 5.0–16.3 yrs)/>2 yrs.	Self-reports: FES, DSP, and study-specific questionnaire measuring parents' concerns.	Family environment and personal stress did not differ between groups and was within 1 SD from norms. Mothers of CCSs scored lower than the comparison group on two subscales on FES; 'intellectual and cultural orientation' and 'recreational activity' (t = 2.99, p<0.01; t = 2.81, p = 0.01). More mothers of CCSs (78%) than mothers in the comparison group reported feeling anxious when their child developed a routine illness (43%) (χ^2^ = 26.03, p<0.001).	NA
Greenberg [30], (1991) USA	Qualitative and cross-sectional.	N = 120 (gender NR).	Mixed	M = 3.6 yrs (range = 2 mths-9 yrs).	M = 12.4 yrs	M = 8.8 yrs (range = 5.0–16.3 yrs)/>2 yrs.	Interviews based on the questions: "What got you through your child's illness?" and "How do you feel about the difficult experience?".	Responses categorized as positive and/or negative. Positive responses: importance of a good social support systems, changed values and attitudes, and positive marital adjustment. Negative responses: concerns regarding late-effects and the child's health, marital problems in terms of inability to meet each other's emotional needs, and feelings of guilt and anger.	NA
Hardy [38], (2008) USA	Quantitative, cross-sectional, and comparative. Comparison group: parents of children on active cancer treatment.	N = 27 parents of CCSs (24 mothers/3 fathers). Comparison group: 28 parents (25 mothers/3 fathers).	Mixed	M = 7.1 yrs (SD = 5.3 yrs) Comparison group: M = 8.3 yrs (SD = 4.7 yrs).	M = 25.6 yrs (SD = 4.0 yrs) Comparison group: M = 9.4 yrs (SD = 4.2 yrs).	M = 18.4 yrs (SD = 7.0 yrs)/NR Comparison group: M = 1.0 yr (SD = 1.5 yrs)/NR.	Self-reports: BSI, IES, PECI, IFS, CGSQ.	Overall similar ratings of parents of CCSs and parents of children on cancer treatment. Both groups reported general psychological distress within a normal range, elevated level of PTSS, persistent worry about the child's health and well-being, uncertainty about the child's future, and intrusive thoughts about the child's cancer experience. Parents of CCSs reported a lower level of caregiver burden (t = 4.27, p<0.001, 95% CI: 0.40–1.11), and more unresolved anger and sorrow (t = 2.60, p<0.017, 95% CI: 0.16–1.22). 44% of parents of CCSs reported a high level of PTSS.	NA
Kazak [37], (1994) USA	Quantitative and longitudinal. Measurement at T1 and T2 (T2 = 1 yr after T1).	T1: N = 74 (71 mothers/3 fathers) T2: N = 59 (gender NR).	Mixed	M = 3.7 yrs.	T1: M = 12.3 yrs (range = 10–15 yrs).	T1: M = 8.0 yrs (SD = 2.5 yrs)/>5 yrs.	Self-reports: LSC, FACES-III.	At group-level psychological distress within norms at T1 and T2. At T1 and T2 20–30% scored indicative of searching help for psychological distress on the LSC and 8.8–10.0% indicative of significant psychological distress. Family functioning within norms.	NA
Kupst [32], (1995) USA	Quantitative and longitudinal, T1–T4. (T1 at Dx, T2 = 2 yrs post Dx, T3 = 6 yrs post Dx, T4 = 10 yrs post-treatment).	T4: N = 35 (23 mothers/12 fathers).	Leukemia all types	M = 6.5 yrs (SD = 4.5 yrs).	T4: M = 19.1 yrs (SD = 4.5 yrs, range = 14–30 yrs).	T4: M = 13.3 yrs (SD = 1.0 yr)/M = 10 yrs (SD = NR).	Self-reports: CARS, BSI, WCS. Social/Ecological variables using the Hollingshead two-factor index. Semi-structured interview staff-rated according to the FCS to assess family coping (parents and children).	Parents were well adjusted to life according to self- and staff-ratings; CARS (>6) and FCS (>30). Psychological distress within norms (GSI, T-score M = 51.91, SD = 15.50). A composite measure (low scores on CARS, FCS and high GSI) classified 18% of families as poor copers.	SES related to mothers' coping adequacy (r = −0.66, p<0.001). Child's coping adequacy and perceived adjustment 10 yrs after Dx predicted mothers' coping adequacy and adjustment (F-change = 8.95, p<0.01; F-change = 57.3, p<0.001). Mothers' coping at Dx predicted mothers' coping adequacy and perceived adjustment 10 yrs after Dx (F-change = 19.83, p<0.001).
Kupst [31], (1988) USA	Quantitative and longitudinal, T1–T3. (T1 = at Dx, T2 = 2 yrs post Dx, T3 = 6 yrs post Dx).	N = 43 (25 mothers/18 fathers).	Leukemia all types	M = 6.5 yrs (SD = 4.5 yrs).	M = 12.8 yrs (SD = 4.5 yrs).	M = 6.8 yrs (SD = 1.5 yrs, range = 6-8 yrs)/NR.	Self-reports: CARS. Semi-structured interview staff-rated according to the FCS to assess family coping (parents and children).	Parents were well adjusted to life according to mean values in the CARS (>6) and FCS (>30).	Not reported for parents of survivors isolated from parents of diseased and relapsed children.
Leventhal-Belfer [35], (1993) USA	Quantitative and cross-sectional.	N = 37 (24 mothers/13 fathers).	Mixed	Birth to adolescence	Median = 20 yrs (range = 8–36 yrs)	Median 11 yrs (range = 6–35 yrs)/NR	Self-reports: SCL-90, PCCSQ, Locke-Wallace Short Marital Adjustment Test.	Reported areas of concerns: child's health complications (91.7% of mothers; 100% of fathers); desire for increased professional support (62.5% of mothers, 38.5% of fathers); relapse (50.0% of mothers; 61.5% of fathers); child's social development (41.7% of mothers, 53.8% of fathers); child's death (16.7% of mothers and 23.1% of fathers).	Mothers of boys perceived their son's cancer history as having a greater impact on them than did mothers of girls (p≤0.02).
Maurice-Stam [39], (2008) The Netherlands	Quantitative and longitudinal, T1–T6 at different times after end of treatment. (T1 = 2 mths, T2 = 1 yr, T3 = 2 yrs, T4 = 3 yrs, T5 = 4 yrs, T6 = 5 yrs).	T3: N = 185 (98 mothers/87 fathers), T6: N = 43 (24 mothers/19 fathers).	Mixed	M = 6.8 yrs (SD = 4.5 yrs; range = 0.3–17.2 yrs).	T1: M = 8.0 yrs (SD = 4.5 yrs).	T1: M = 13.7 mths (SD = 8.4)/2 mths.	Self-reports: GHQ-30, SSERQ, CCSS-PF, UCL, FACES, SSQT, and medical variables (e.g. prognosis, treatment intensity).	From 2 yrs after end of treatment normal levels of psychological distress. Frequency of disease related feelings stable from 2 yrs after end of treatment, except that fathers reported fewer positive feelings 3 yrs after end of treatment compared to directly after treatment (p<0.05), and fewer feelings of loneliness 4 yrs after end of treatment compared to directly after end of treatment (p<0.01).	Predictors measured at T1. Disease-related coping predicted feelings of uncertainty (est = −0.33, p<0.01) and helplessness (est = −0.32, p<0.01). Longer duration of treatment (est = −0.14, p<0.01) and greater optimism about the course of the disease (est = −0.17, p<0.01) associated with lower levels of distress. Passive reaction pattern associated to higher levels of distress (est = 0.36, p<0.01).
Michel [12], (2010) UK	Quantitative and cross-sectional.	N = 45 (40 mothers/5 fathers).	Mixed	M = 5.0 yrs (SD = 3.0 yrs; range 0.2–11.5 yrs).	M = 13.7 yrs (SD = 1.1 yrs; range 12.0–15.9 yrs).	NR/M = 6.6 yrs (SD = 2.9 yrs; range = 2.9–13.9 yrs).	Self-reports: PTGI SF-12, PCL-C, IPQ.	PTGI, M = 2.7(CI: 2.4–3.0), SF-12; PCS, M = 53.2 (CI: 49.6–56.9), MCS, M = 46.3 (CI: 43.2–49.4), PCL-C, M = 31.1 (CI: 26.8–35.4). No comparison with norms reported.	Perceptions of how much the illness still affects them emotionally associated with PTG (B = 2.3, p = 0.02).
Overholser [42], (1991) USA	Quantitative and cross-sectional.	N = 44 (gender NR).	Mixed	M = 10.3 yrs (SD = 4.0 yrs).	NR	NR/2–7 yrs	Staff-ratings via structured interviews assessing parents' perceptions of social support, family functioning, and global adjustment. Self-reports: IFS.	IFS: Financial burden 9.89 (SD = 3.45), Familial/Social support 20.21 (SD = 5.22), Personal Stain 14.96 (SD = 3.08), Mastery 10.04 (SD = 2.51). No comparison with norms reported.	Personal strain related to parental tendency to express anger towards the child. Low mastery score associated with negative views of the child. High mastery score associated with social support during child's illness. Increased mastery in parents related to better long-term global adjustment in the child. Setbacks in the mothers work role. Independent variables drawn from self-ratings and dependent variables from answers in structured interviews and clinical ratings of these.
Ozono [34], (2010) Japan	Quantitative and cross-sectional.	N = 159 (87 mothers/72 fathers).	Mixed, brain tumors excluded.	M = 5.4 yrs (SD = 3.8 yrs).	16.2 yrs (SD = 2.2 yrs; range = 12–20 yrs).	>5 yrs, M = 10.8 yrs (SD = .4 yrs)/>1 yr.	Self-reports: IES-R, ZSDS, STAI, FRI	Levels of PTSS, depression and anxiety below clinical significance.	Splits sample based on FRI ratings and categorizes individuals based on family functioning in three clusters; "supportive type", "intermediate type", and "conflictive type". Conflictive type significantly higher levels of PTSS compared to supportive and intermediate type. Lowest levels of depression in parents in the supportive cluster. Supportive lowest scores on STAI, conflictive type highest STAI score. PTSS associated with chaotic family functioning.
Ozono [33], (2007) Japan	Quantitative and cross-sectional.	N = 159 (87 mothers/72 fathers).	Mixed, brain tumors excluded.	M = 5.4 yrs (SD = 3.8 yrs).	16.2 yrs (SD = 2.3 yrs; range = 12–20 yrs).	>5 yrs, M = 10.8 yrs (SD = 3.4 yrs)/>1 yr.	Self-reports: IES-R, STAI, FAD, Holmes-Rahe measure of social adjustment.	Severe PTSS among 21% of mothers and 22% of fathers.	Higher trait (*B* = 0.12, p<0.01) and less than 10 yrs since Dx (*B* = 1.86, p<0.01) significant factors for severe PTSS among mothers. Among fathers trait anxiety was a significant factor for severe PTSS (*B* = 0.07, p<0.05). Associations between levels of PTSS for mothers-survivors (r = 0.377, p<0.01) and mothers-fathers (r = 0.483, p<0.01).
Quin [36], (2005) Ireland	Quantitative/qualitative and cross-sectional.	N = 120 (74 mothers/46 fathers).	Mixed	NR	M = 12 yrs (range 3–21 yrs).	NR/>2 yrs	Self-reports: COPE, GHQ, In-depth interviews.	Normal levels of psychological distress, GHQ M = 18.4. Average levels of coping compared to norms. Higher levels on seeking comfort through involvement in religious activities than norms. Emerging themes: Experiences of diagnosis and treatment; perceived changes in the child; effects on the family as a whole, on the marital relationship, on the child and on their siblings; current perceptions about the ongoing effects on the child, and anticipations for their future.	Of those scoring low on both COPE and GHQ all were mothers. Mothers made greater use of many coping strategies, internal and external, than did fathers. Fathers tended to rely on mental disengagement, denial, and use of alcohol/drugs.
Stuber [41], (1996) USA	Quantitative and cross-sectional.	N = 105 (63 mothers/42 fathers)	Leukemia, all types	M = 4.6 yrs (SD = 3.2 yrs; range 1–13 yrs)	14.0 yrs (SD = 3.2 yrs; range 7–19 yrs)	NR/>2 yrs, M = 6.7 yrs (SD = 2.8 yrs)	Self-reports: PTSDRI	39.7% of mothers and 33.3% of fathers reported a severe level of PTSS. The majority of symptoms were classified as re-experiencing/intrusive symptoms, some symptoms as avoidance/numbing, no symptom of arousal was reported.	Mothers' and survivors' PTSS scores associated (r = .29, p<0.05), as mothers' and fathers' (r = .41, p<0.01). Time since Dx not related to PTSS.
Wijnberg-Williams [40], (2006) The Netherlands	Quantitative and longitudinal, T1–T4. (T1 = at Dx, T2 = 6 mths after Dx, T3 = 1 yr after Dx, T4 = 5 yrs after Dx). At T4: comparison made with parents of relapsed and deceased children in the cohort.	N = 86 (gender NR).	Mixed	M = 5.7 yrs (SD = 4.6 yrs; range 0–16 yrs).	T1:<15 yrs	T4: 5 yrs/NR	Self-reports: GHQ, SCL-90, STAI-S.	T4: Clinically elevated scores of general distress among 23% of parents measured with the GHQ. STAI and SCL-90 within normal range. Parents of survivors reported significantly lower levels of anxiety than parents of deceased children (U = 614.50; p = 0.023). No difference between groups regarding SCL-90 and GHQ.	NA

*Note.* BSI = Brief Symptom Inventory; CARS =  The Current Adjustment Rating Scale; CCSS-PF = Cognitive Control Strategies Scale for Parents; CGSQ = Caregiver Strain Questionnaire; COPE = Cope-scale; CPI =  California Psychological Inventory; DSP = Derogatis Stress Profile; FACES-III =  The Family Adaptation and Cohesion Evaluation Scales-Version-III; FAD =  The Family Assessment Device; FCS =  Family Coping Scale; FES = Family Environment Scale; FRI = The Family Relationship Index; GHQ = General Health Questionnaire; GSI = Global Severity Index; IES = Impact of Event Scale; IES-R =  The Impact of Event Scale-Revised; IFS = Impact on Family Scale = ; IPQ = The Brief Illness Perception Questionnaire; LSC = The Lagner Symptoms Checklist; MCS = Medical Component Score; PCCSQ =  Parents of Childhood Cancer Survivors Questionnaire; PCL-C = The PTSD Checklist-Civilian Version; PCS = Physical Component Score; PECI = Parent Experience of Child Illness Scale; PTGI = Post Traumatic Growth Inventory; PTSDRI =  Posttraumatic Stress Disorder Reaction Index; SCL-90 = Symptom Check List-Revised; SF-12/-36 = Short Form Health Survey-12/-36; SSERQ = Situation-Specific Emotional Reaction Questionnaire; SSQT =  Social Support Questionnaire for Transaction; STAI = State-Trait Anxiety Inventory; UCL = Utrecht Coping List; WCS = Ways of Coping Scale; ZSDS = The Zung Self-Ratings Depression.

Dx = diagnosis; NA = not applicable; NR =  not reported; SES = socioeconomic status.

a Statistical values reported if reported in the study.

### Assessment of Quality

Results from the quality assessment of studies using quantitative methodology are presented in Table 1, and of studies using qualitative methodology in Table 2. The quality of the study using both quantitative and qualitative methodology was assessed twice. Three of the 14 studies using quantitative methodology were assessed as of overall low quality, six as of overall moderate quality, and five as of overall high quality. To assess risk of bias across studies, scores were summarized for each of the 12 assessed items. Three items were assessed as of low quality, four as of moderate quality, and five as of high quality. Items regarding response rate, comparison of responders and non-responders, and sample size was rated as of low quality. These items are related to risk of selection bias. Study aim, study design and method, robustness of outcome measurements, and conclusions supported by results were rated as of high quality. One of the studies using qualitative methodology was assessed as of moderate and one as of low quality. Items regarding analytic method, use of verification process, and reflexivity of the account were assessed as of low quality in the studies using qualitative methodology.

### Synthesis of Results

The studies using quantitative methodology reported on the following: general psychiatric symptoms/psychological distress (9 studies), family functioning (6), concerns and caregiver strains (4), coping (3), PTSS (3), anxiety (1), marital adjustment (1), and situation-specific emotions (1). Two studies reported on factors associated to psychological late effects, however not on the prevalence of the phenomena per se. The studies using qualitative methodology reported on themes such as family functioning, marital adjustment, positive change, and worry. Results were summarized and presented as negative long-term psychological late effects, general long-term psychosocial adjustment, and positive long-term psychological late effects. In 10 studies factors associated with/predicting long-term psychological late effects were reported.

### Negative Long-term Psychological Late Effects

#### General psychiatric symptoms and psychological distress

General psychiatric symptoms and psychological distress were generally found to be comparable to normative samples. Leventhal-Belfer et al. [35], Kazak et al. [37], Kupst et al. [32], Quin [36], Hardy et al. [38], and Maurice-Stam et al. [39] reported a group-level of psychological distress within a normal range. Greenberg et al. [29] compared personal stress among mothers of CCSs and mothers of non-ill children and found no difference between groups. Wijnberg-Williams et al. [40] reported a group-level of anxiety within a normal range and Ozono et al. [34] reported group-levels of anxiety and depression below clinical significance. However, Wijnberg-Williams et al. [40] found that 23%, compared to 15% in a norm-group, scored above a cut-off indicative of a clinically relevant level of psychological distress. Similiarly, Kazak et al. [37] showed that 20–30% reported psychological distress indicative of seeking help and that 8.8–10.0% reported significant psychological distress.

#### PTSS

Subgroups with a clinically relevant level of PTSS were identified in all studies reporting on PTSS. Ozono et al. [34] demonstrated an average group-level of PTSS, but that a subgroup, 21% of mothers and 22% of fathers, reported a severe level of PTSS [33]. Stuber et al. [41] found that 40% of mothers and 33% of fathers reported a severe level of PTSS whereas Hardy et al. [38] showed that 44% reported a high level of PTSS. Stuber et al. [41] classified the majority of the PTSS symptoms as re-experiencing or intrusive symptoms and showed that more than 75% of mothers reported: being afraid or upset when thinking of what had happened, tension when thinking of cancer, re-experiencing disturbing scenes, intrusive thoughts of the cancer disease, distress at reminders, and fear of relapse. More than 75% of the fathers reported being afraid or upset when thinking of cancer, and tension when thinking of what had happened.

#### Worry

When interviewed in the study by Greenberg and Meadows [30] 49% started the interview by discussing their concerns for their child, and almost 75% mentioned that their main concern was the treatment's late effects on their child. Other areas of concern were the child's school performance, lack of friends, lack of growth, and possible infertility. Leventhal-Belfer et al. [35] reported that parents' most frequent concern was the child's health and potential complications related to the cancer treatment. This result was supported by findings by Quin [36] showing that almost one-third expressed worries about some or all aspects of their child's health. In addition concerns about the impact of the illness on siblings, and the child's future regarding e.g. personal relationships, education, and employment were expressed. Parents in the study by Quin [36] reported having persistent feelings of fear, worry, and insecurity:

“You're not as secure in your life. You realize that things can go very wrong”. [36]

Greenberg et al. [29] found that mothers of CCSs were more likely than comparison mothers to report feeling anxious when their child developed a routine illness. In the study by Leventhal-Belfer et al. [35] the majority reported fear of relapse, however only a minority reported fearing that the child would die as a result of the relapse; 17% of the mothers and 23% of the fathers mentioned this concern. Hardy et al. [38] demonstrated that parents reported persistent worry about their child's health and well-being, uncertainty about their child's future, and intrusive thoughts about the cancer experience.

#### Disease-related thoughts and feelings

When interviewed in the study by Greenberg and Meadows [30], 13% of the parents mentioned persistent feelings of guilt and/or anger and when interviewed in the study by Quin [36] guilt, self-blame, and anger related to the child's previous cancer disease was reported. Hardy et al. [38] found that parents of CCSs reported higher levels of unresolved anger, more sorrow associated with the loss of the healthy child they had before the illness, and more jealousy towards families that had not experienced the hardship associated with the child's disease, than parents of children on active treatment for cancer. Maurice-Stam et al. [39] demonstrated that helplessness and uncertainty related to the child's disease decreased during the first year after end of treatment, and returned to normal level two years after end of treatment.

### General Long-term Psychosocial Adjustment

#### Adjustment and coping adequacy

In the study by Greenberg et al. [29] mothers reported lower levels of “intellectual and cultural orientation” and “recreational activity” than mothers of children not struck by a serious disease. Parents reported positive/adequate adjustment and coping [31], [32] and these findings were supported by results by Quin [36] demonstrating levels of coping comparable to norms. Quin [36] also demonstrated that parents of CCSs seek more comfort through involvement in religious activities and experience less social support than norms.

#### Family functioning

Greenberg et al. [29] demonstrated that mothers of CCSs and mothers of children not struck by a serious disease reported equal, and average, levels of family environment, i.e. satisfaction with relationships, personal growth, and system maintenance. These results were supported by findings by Kazak et al. [37] showing levels of family functioning within norms. Hardy et al. [38] showed that parents of CCSs reported a lower impact of illness on family functioning and a lower level of caregiver burden than parents of children on active treatment for cancer.

When interviewed in the study by Quin [36] parents reported that the cancer experience had both positive and negative effects on the family. One third regarded the experience as having an overall positive effect on family relationships in the sense of becoming closer, living more in the present, and being less preoccupied with material things. Approximately one fourth perceived the experience as having an overall negative effect on the family, e.g., that the focus had been on the sick child to the extent that siblings had suffered and the marital relationship deteriorated. The remaining parents mentioned that the illness, while very traumatic at the time, had no persisting effects on the family.

#### Marital adjustment

In the study by Greenberg and Meadows [30] most parents did not mention marital adjustment as an issue, 23% mentioned that the marriage had been positively impacted by the child's illness while 25% reported that they experienced marital difficulties, e.g., not being able to meet each other's emotional needs. In the study by Leventahl-Belfer et al. [35] parents reported a relatively high level of satisfaction regarding communication with the partner. In the study by Quin [36] almost a fourth mentioned that the cancer experience had some negative impact on their couple relationship while a small minority reported that the strain on their marriage was intense. The vast majority of the parents, almost three-quarters, mentioned that their relationship was strengthened as a result of their child's illness:

“I'd say it definitely grew us closer in our marriage. We just realized we needed each other to get through it”. [36]

### Positive Long-term Psychological Late Effects

When interviewed in the study by Greenberg and Meadows [30] 26% reported consequences such as “a growth experience”, “being tougher”, and “seeing what is really important in life”. In the study by Quin [36] almost four-fifths of the fathers mentioned that the experience had affected them in positive ways, most often through changed perspectives and priorities. Parents expressed that the experience had made them more patient and attentive towards their children and that it had made them “softer”. However, parents also reported being more over-protective and having a tendency to “spoil” their child. Among mothers the most commonly described positive late effect was a changed life-perspective:

“It changed my whole attitude to life, to be honest. You realize how precious life is”. [36]

### Factors Associated with/Predicting Long-term Psychological Late Effects

#### Time since diagnosis

Kupst and Schulman [31] did not find an association between time since diagnosis and adjustment, and Stuber et al. [41] did not find an association between time since diagnosis and level of PTSS. However, Ozono et al. [33] reported that less time (<10 years) had passed since the child's diagnosis for mothers who reported a severe level of PTSS.

#### Child's age at diagnosis

Leventhal-Belfer et al. [35] did not find an association between child's age at diagnosis and parents' level of mental health and/or adaptation as a couple, and Stuber et al. [41] did not find an association between child's age at diagnosis and parents' level of PTSS. In contrast Ozono et al. [33] found that mothers of children diagnosed at a higher age reported a higher level of PTSS.

#### Parental gender

Leventhal-Belfer et al. [35] demonstrated an interaction effect between parents' and children's gender with mothers of boys perceiving their son's cancer history as having a greater impact on them than mothers of girls. No such interaction was shown for fathers. Stuber et al. [41] demonstrated that mothers' level of PTSS was within a moderate range while fathers' level of PTSS was within a mild range. Quin [36] found that the individuals reporting the lowest level of general health and coping adequacy were mothers. In the same study fathers reported a higher level of mental disengagement, denial, and use of alcohol/drugs than mothers.

#### Social support

In the study by Overholser and Fritz [42] high perceived social support, instrumental and emotional, during the child's illness was related to high perceived mastery. In the study by Greenberg and Meadows [30] parents were asked what had got them through their child's disease, and 51% reported that they had been helped by good social systems such as their families, hospital staff, the church, community groups, and other parents.

#### Family functioning

In the study by Ozono et al. [34] parents in conflictive families reported the highest level of depression, anxiety, and PTSS. Cohesive families dealt well with life after cancer, supported each other, shared emotions, and reported mutual caring and lower levels of depressive symptoms than parents in conflictive families.

#### Reminders of the disease

In the study by Maurice-Stam et al. [39] parents of children without visible late effects of the disease and treatment reported a lower level of disease-related helplessness than parents of children with visible late effects. Michel et al. [12] found a positive association between parents' perceptions of the degree that the past affects them emotionally today, and the current level of PTG.

#### Child's adjustment

Kupst et al. [32] found that the child's current coping and adjustment was associated with parental current coping adequacy. In the study by Stuber et al. [41] mothers' and fathers' as well as mothers' and CCSs' levels of PTSS were associated (CCSs' PTSS rated by clinicians). No association was found between fathers' and CCSs' reports. The same pattern was found by Ozono [33] reporting associations between mothers' and CCSs', and mothers' and fathers' reports of PTSS, while no association was found between fathers' and CCSs' reports. Michel et al. [12] found no association between children's levels of benefit findings and parents' levels of PTG.

#### Previous parental adjustment

Kupst et al. [32] demonstrated that mothers' adjustment adequacy at diagnosis and two years after diagnosis predicted later coping adequacy. This corresponds with results by Maurice-Stam et al. [39] showing that an active problem focus and comforting cognitions shortly after end of treatment was related to positive feelings five years after treatment, while more passive reaction patterns was related to psychological distress. Compared to medical and demographic characteristics, parents' previous coping was a stronger predictor of emotional function 10 years after diagnosis.

## Discussion

### Summary and Clinical Implications

The overall objective was to describe the nature and prevalence of long-term psychological late effects, i.e. present at least five years since the child's diagnosis and/or two years since end of treatment, for parents of CCSs. To the best of our knowledge, this is the first systematic review investigating these effects.

Fifteen studies, published between 1989 and 2010, met the inclusion criteria. Even though cancer therapies have changed dramatically during these years [43], results were strikingly similar across studies. At group-level parents reported general psychological distress within a normal range in all nine studies reporting on general distress. Levels within a normal range were also reported regarding family functioning and coping. However, subgroups reporting clinically relevant levels of general psychological distress and PTSS, respectively, were identified; 21–44% of the parents reported PTSS at a severe level. These figures correspond with results for parents of children during earlier stages of the cancer-disease trajectory [7], [44], with other chronic illnesses [45], and pediatric burns [46].

The systematic review by Bruce [7] reported severe PTSS among 10–44% of parents of CCSs. The high prevalence of severe PTSS identified in this review indicates that a substantial subgroup of parents experience severe distress even when at least five years have passed since the child's diagnosis and/or two years since end of the treatment. This finding agrees with recent results for survivors of childhood [6] and adult cancer [24] showing that a subgroup of survivors is at risk for psychological distress even 10 years or more after diagnosis. Brinkman et al. [6] also reported that a subgroup is at risk for persistent or even increasing psychological distress decades after the cancer diagnosis. The results from this review are the first to suggest a similar pattern for parents of CCSs.

To map and understand the nature of the long-term psychological late effects that parents of CCSs experience we identified all aspects of psychological functioning and adjustment, not only specific symptom clusters and psychiatric diagnoses. Aside from psychiatric symptoms parents reported anger, guilt, self-blame, and fear of relapse. One of the reviewed studies showed that parents of CCSs reported more anxiety in response to children's routine illnesses compared to parents of children not diagnosed with cancer [29]. Given that a substantial subgroup of parents reported a clinically relevant level of PTSS, the existence of intrusive thoughts and anxiety in response to stimuli reminding of the child's cancer disease is not surprising. Several of the reviewed studies reported worries and concerns regarding the child's health, social life, and possible infertility. Stuber et al. [41] found intrusive thoughts to be the dominating PTSS symptom among parents of CCSs. Considering treatments for this population interventions directed at PTSS could be advantageous and possibly also have an effect on symptoms such as disease-related thoughts and feelings, and worry.

The majority of the participants in the reviewed studies did not report that the marital relationship was negatively affected by the cancer experience. Some reported that their relationship was strengthened as a result of the experience, but a subgroup reported marital difficulties. These results correspond with those for parents of children during earlier phases of the child's cancer disease trajectory [47]. The results from this review indicate that for some parents, negative effects on the marital relationship can persist over a long time and may be important to address in psychological treatments for this population.

In the qualitative studies long-term positive late effects were reported. Improved relationships and changed values due to the cancer experience were reported years after diagnosis and/or end of successful treatment. However, a limitation is that positive consequences were not assessed in the quantitative studies with the exception of one [12], and that study did not report on the prevalence of perceived positive consequences.

Ten studies reported on factors associated with or predicting long-term psychological late effects and some consistencies were found between these studies. Parents' coping and adjustment were stronger predictors of parents' emotional function than children's medical and disease-related variables [32], [39]. This underscores the importance of supporting parents in earlier time-phases of the child's disease. When interviewed, parents reported social support as having got them through the child's illness [30], [42]. Facilitating access to social support systems can be one important aspect of psychological help to parents of children on cancer treatment.

Parents of children without visible physical late effects reported less disease-related helplessness [39]. This finding agrees with results showing that children's physical late effects are related to parents' levels of PTSS [48] and that permanent scaring among children with burn damage is related to severe PTSS among parents [49]. As previously argued by Norberg and Green [50], these findings indicate that distress in parents of children struck by somatic disease/physical damage is not entirely related to past events, but also to current disease-related stressors.

### Assessment of Quality

The strength of evidence was evaluated through an assessment of the quality of reviewed studies. Three studies using quantitative and one study using qualitative methodology were assessed as of overall low quality. These studies were all conducted before 1994 and were thus among the oldest of the reviewed studies. Since the literature concerning long-term psychological late effects of childhood cancer for parents of CCSs is limited we chose to include all identified studies in the review in spite of their quality. Three items, response-rate, description of non-responders/comparison of responders and non-responders, and sample size concerning studies with quantitative methodology got a total quality score indicating low quality. This indicates that a risk of selection bias in the reviewed studies using quantitative methodology must be considered. It should however be taken into consideration that the population of interest is small and that small sample sizes are to be expected.

### Limitations of the Current Study

There are a number of limitations that need to be addressed. Firstly, the search terms used were broad in order to capture as many aspects as possible of the long-term cancer-related psychological late effects that parents of CCSs may experience. Despite this broad strategy only 15 studies met the inclusion criteria and these were diverse in several respects. This precluded from any possibility of conducting a meta-analysis which would have given a more direct estimate of the prevalence or risk of a late effect. Secondly, the search was limited to research published in English language journals which might have resulted in studies published in other languages being left out. The majority of the studies included in this review were also conducted in English speaking countries which may limit the generalizability of the findings. Finally, there were more mothers than fathers included in the studies reviewed which may hamper the generalizability of the findings.

### Recommendations for Future Research

Important implications have been derived from the current review which can improve the quality of future research in this field. We identified poor quality in studies using quantitative methodology with regard to response-rate, sample size, and description of responders and non-responders; all of which are related to risk of selection bias. It is of high importance that future research in this area put effort into recruiting large, representative samples. Parents of children with cancer are per se a limited population which warrants national, as well as international, collaborations. Quality assessments of qualitative studies point to the need of improved quality with regard to e.g. systematic analyses and verification procedures. Further it is worth to notice that the vast majority of reviewed studies used a quantitative methodology. To explore, describe, and understand the suffering in the population at hand qualitative research can contribute with important information possibly omitted in previous quantitative research. Such research could be used to develop population specific questionnaires which would help to better understand development of distress in this population over long-term.

The overall finding of this systematic review is that there is a substantial subgroup of parents of CCSs who report long-term psychological late effects. An increased understanding of the development and maintenance of this distress is warranted. Such an understanding can illuminate to what extent the distress is related to current stressors such as medical late effect experienced by the previously ill child, and/or whether the distress is a manifestation of parents' psychological reactions to adverse events during the time when the child was ill and under treatment. Future studies should aim at providing a theoretical understanding of the development and maintenance of the cancer-related distress experienced by parents of CCSs.

In addition, future research should explore and describe the positive cancer-related psychological late effects experienced by parents of CCSs. None of the reviewed quantitative studies investigated this phenomenon. On the basis of inductively derived knowledge regarding the cancer-related suffering and positive consequences experienced by CCSs questionnaires measuring these aspects should be developed and used in future research. Due to the low number of studies matching the inclusion criteria we chose to include old studies and studies assessed as of low quality in the present systematic review. When more research has been conducted in the area of this systematic review we suggest that an update of this review with a narrower time-span is done.

As previously addressed, to this date there is no evidence based psychological treatment for the subgroup of parents of CCSs who experience cancer-related suffering. Future research should focus on developing a psychological treatment for the population at hand.

### Conclusions

This review has identified methodological weaknesses in the research on long-term psychological late effects of childhood cancer for parents. This, as well as the low number of studies in the area, precludes firm conclusions with regard to the long-term psychological late effects of childhood cancer for parents. Taking this into consideration the findings suggest that at group level parents of CCSs at least five years post diagnosis and/or at least two years off treatment experience levels of psychological distress, coping, and family functioning that are within a normal range. However, a substantial subgroup reports a clinically relevant level of general psychological distress and/or PTSS, and expresses worry, fear of relapse, anger, and/or sorrow related to the child's previous disease. Some parents also report post-traumatic growth. Taking these results into consideration, it could be motivated for the clinical care to routinely screen for psychological distress among parents of CCSs. To date there is no evidence-based psychological treatment to offer these parents. It is suggested that psychological treatments targeting PTSS, and possibly worry, anger, sorrow, and marital problems could be beneficial for parents of CCSs.

## Supporting Information

Checklist S1 **PRISMA Checklist for Systematic Reviews.**
(DOC)
